# Associated Factors of Functional Ability in Older Persons Undergoing Hip Surgery Immediately Post-Hospital Discharge: A Prospective Study

**DOI:** 10.3390/jcm12196258

**Published:** 2023-09-28

**Authors:** Kanokwan Monkuntod, Suparb Aree-Ue, Inthira Roopsawang

**Affiliations:** Ramathibodi School of Nursing, Faculty of Medicine Ramathibodi Hospital, Mahidol University, Bangkok 10400, Thailand; kanokwan.mou@mahidol.ac.th (K.M.); inthira.ros@mahidol.edu (I.R.)

**Keywords:** adverse health outcomes, functional ability, hip fracture surgery, older people

## Abstract

Background: hip fractures commonly have an impact on older adults’ health. Surgical treatment aims to reduce pain and promote functional ability. However, developing adverse health outcomes or complications post-hip surgery may impede older patients’ recovery to return to functional ability as pre-fracture. We aimed to examine the association of personal factors and adverse health outcomes during hospitalization and post-hospital discharge on the functional ability of older people undergoing hip surgery. Methods: a total of 120 older people with hip fractures who were scheduled for surgery at three tertiary hospitals and met the inclusion criteria were recruited for this study. Data were obtained at admission, before discharge, and during the two-week postoperative follow-up using the Demographic, Hip Dysfunction and Osteoarthritis Outcome Score, Joint Replacement, the Confusion Assessment Method (CAM) Thai version, and Health Outcome Questionnaires. Descriptive statistics and multiple logistic regression analyses were performed to analyze the data. Results: most participants were female, with a mean age of 78.10 years (range = 60–93; SD = 8.37). The most common adverse health outcome during hospitalization was urinary tract infection, followed by delirium, pneumonia, deep vein thrombosis, and surgical site infection. At two weeks immediately post-hospital discharge, 16 participants experienced unpleasant events, including delirium, urinary tract infection, surgical site infection, and pneumonia. The significant predictors of poor functional ability at two weeks immediately post-hospital discharge were old age (OR = 1.114, *p* = 0.001), subtrochanteric fracture (OR = 13.48, *p* = 0.008), and type of surgery (OR = 4.105, *p* = 0.049).

## 1. Introduction

Hip fracture due to falls is a common problem leading to life-threatening consequences in older adults, particularly those with advanced age or osteoporosis [1]. The global prevalence of hip fracture has increased sharply since 1990 [2]; the Asian Federation of Osteoporosis Societies (AFOS) demonstrated an upsurge to 2.56 million by 2050, showing an increase of 2.28-fold compared to 2018 [3]. Similar to other countries, the incidence rates for hip fractures in Northern Thailand were reported to be increasing, with a higher incidence in women than men (ratio, 2.5:1) [4]. Hip fracture is known to have an impact on the quality of life resulting from limitations in performing activities of daily living (ADL), self-care, and mobility, as well as participating in social activities [5,6]. In addition, hip fractures among older people cause them to become dependent and lose their mobility. The Barthel Index assessing the ability to perform activities of daily living fell from 79 to 44% after sustaining the fracture [7]. Moreover, hip fractures affect not only the patients themselves but their relatives and caregivers, as well resulting in problems with depression, relationships, and finances [8]. These multidimensional impacts require comprehensive and continuous treatment and specific care. Hip fracture treatment can involve both surgical and non-surgical methods depending on the patients’ health conditions [9,10].

Although hip surgery is expected to improve functional recovery to pre-fracture levels, there is a risk of developing various postoperative complications, especially in older people during hospitalization or post-hospital discharge. Common complications—surgical site infections, hip dislocations, peri-prosthetic fractures or infections, pneumonia, urinary tract infections (UTIs), delirium, and deep vein thrombosis (DVT)—are prevalent [11,12,13,14]. These are the primary adverse health outcomes that can mostly occur during hospitalization or immediately post-discharge, leading to decreased functional ability and long-term dependency postoperatively. Moreover, a previous study indicated that many factors for optimizing functional ability include age, gender, comorbidities, types of surgery, and adverse health outcomes; the patients treated with cemented hemiarthroplasty and proximal femoral nailing are different in the functional ability for three months [15]. Therefore, effective management and timely functional assessment are essential for functional restoration in this population.

Usually, after being discharged home post-hip surgery, the patient may need a two-week follow-up—an immediate post-surgery period—to assess unfavorable health outcomes and functional ability—an indicator for functional recovery post-hip surgery—so that they can be remedied promptly to prevent long-term adverse health outcomes and promote regaining functional ability [16,17]. However, information assessing health outcomes and monitoring the functional abilities of older people undergoing hip fracture surgery in the immediate post-surgery period is limited since most studies have employed an intermediate recovery period of six months to one year and contain limited information about the Thai context. Delaying follow-up until the intermediate recovery phase is a remarkably long time and significantly delays assessing or monitoring health outcomes, dramatically increasing the delay in detecting complications that, in turn, may develop into severe, long-term complications. Therefore, we are interested in examining the association of personal factors (age, gender, comorbidities, ASA class, types of fracture, types of surgery) and adverse health outcomes (delirium, pneumonia, surgical site infection, urinary tract infection, and deep vein thrombosis) during hospitalization and post-hospital discharge with functional ability 2 weeks post-hospital discharge in older people undergoing hip surgery. The findings are expected to provide substantial information to raise awareness for planning for, managing, and preventing adverse outcomes related to modifiable factors related to functional ability, ultimately enabling older adults to regain their pre-fracture functionality and to maintain a good quality of life.

## 2. Materials and Methods

### 2.1. Participants and Sample Size

This observational cohort study looked at older people who were diagnosed with a hip fracture, scheduled for hip surgery, and required a two-week postoperative follow-up appointment at three tertiary care hospitals. Simple random sampling was applied for hospital selection; the focus hospital settings were located in the Bangkok metropolitan, Thailand. The participants were 120 older people, and the calculation of the number of participants needed was guided by Burmeister and Aitken’s recommendations [18]. The study participants were enrolled if they met the inclusion criteria: individuals age 60 years and over, with no cognitive impairments, no evidence of hearing loss, able to understand and communicate in the Thai language, without any neuromuscular conditions related to severe walking limitations (e.g., history of cerebrovascular accidents, Parkinson’s disease, or metastatic cancer of the hip, etcetera), who experienced unexpected adverse events that required intensive care or urgent treatment (e.g., pulmonary embolism, septicemia, septic shock, or acute renal failure), and unable to be discharged based on a clinical pathway or referred to other hospitals or nursing home facilities or received any other rehabilitation interventions before follow-up times were excluded from this study.

### 2.2. Measures

A demographic questionnaire was designed to obtain data, such as age, gender, marital status, education level, occupation, income, the sufficiency of income, and types of medical payment. Functional ability was investigated by using the Hip Dysfunction and Osteoarthritis Outcome Score, Joint Replacement (HOOS, JR) [19]. It consisted of six questions with total scores ranging from 0 to 24. The interpretation was conducted by converting it to an interval score ranging from 0 to 100, where 0 points represented total hip disability, while 100 points indicated perfect hip health. The scores of or lower than 48 points (median values of the population) indicated limited functional ability, while the scores higher than 48 points demonstrated good functional ability. A Health Outcomes Questionnaire was developed to record all complications during hospitalization and 2 weeks post-discharge, such as delirium, pneumonia, surgical site infection, deep vein thrombosis, and urinary tract infection. Delirium was evaluated by using the Confusion Assessment Method (CAM) Thai version [20], consisting of four features: (1) the acute onset of mental status changes, (2) fluctuating inattention episodes, (3) disorganized thinking, and (4) alteration of consciousness. The patients who developed features 1 and 2 plus 3 or 4 were classified as delirium. For pneumonia, cough, pleuritic chest pain, shortness of breath, and temperature ≥ 38 °C, an additional chest x-ray is required [21]. Surgical site infection (SSI) was determined based on at least one of the following signs/symptoms: purulent wound discharge, erythema > 1 cm from the wound edge, localized swelling pain, or patient reporting increasing tenderness [22]. Deep vein thrombosis (DVT) was stratified by using Wells’ Criteria for DVT. It includes 10 clinical features: the score −2–0 indicates low risk for DVT; the score 1–2 represents moderate risk; and the score 3-8 means high risk for DVT [23]. In this study, the score ≥ 1 was stratified as unpleasant DVT. Urinary tract infection (UTI) was evaluated using McGeer criteria [24]. Notably, any complications or adverse health outcomes that occurred during admission and 2-week post-hospital discharge would be classified as either present or absent for the final analysis. 

### 2.3. Data Collection

Data were collected between December 2020 and September 2021. The researcher selected the study’s participants using purposive sampling by reviewing the list of patients scheduled for hip surgery in the hospital admission records and medical records daily during the study period. Participants who agreed to participate in the study were asked to sign the informed consent forms. Training by the research team, two registered nurses with more than five years of experience working with older adults conducted data collection processes as research assistants. Demographic data were obtained via an interview before the participants underwent surgery. Complications were assessed daily using various resources to elicit information about patients’ signs and symptoms—from medical records, diagnosis, and laboratory results. Prior to hospital discharge, the researcher made an appointment for a second interview, which was conducted on the date of the participants’ 2-week post-hip surgery follow-up appointment at the Orthopaedic Outpatient Department. However, all concerns about potential complications were monitored every three days for a period of 2 weeks after discharge.

### 2.4. Ethical Considerations

After the Institutional Review Boards of all research settings approval, this study was carried out with consideration to protect the participants’ rights in three areas: risks, benefits or adverse effects, and confidentiality. During the study, the participants were informed that they had the right to withdraw from the study at any time if they felt uncomfortable or inconvenienced while providing information, with no reduction in the level or quality of any treatment they would receive. In addition, to protect the participants’ identities and personal information, data analysis and disclosure were performed using only the information needed for the research and potential information that could identify the participants removed from the files.

### 2.5. Data Analysis

The data were analyzed using the Statistic Package for the Social Science (SPSS) for Windows, version 18 (Licensed Mahidol University). The Pearson product-moment correlation coefficient and Spearman Rank correlation were applied to justify the relationship among variables of interest. Multiple logistic regression analysis was performed to explore the association of the personal factors (gender, age, comorbidities, ASA classification, type of fracture, and type of surgery) and adverse health outcomes (during hospitalization and 2 weeks post-hospital discharge) on functional ability at 2 weeks post-hospital discharge in older adults undergoing hip surgery. Multiple logistic regression tests were used to investigate because the scores for functional ability are skewed in this population; therefore, concerning normalization, the researcher used the median score cut point to classify poor and better functional ability for the analysis. The 0.05% significance level was used in this study to define the results as being statistically significant.

## 3. Results

### 3.1. Demographics and Clinical Status of the Participants

A total of 120 older adults participated in this study. The majority of participants were female (83.3%); the ages ranged from 60 to 93 years old, with a mean age of 78.10 years (SD = 8.37). The majority of the participants (39.2%) had a normal body mass index (BMI of 18.50–22.99 kg/m^2^) and at least one comorbid disease (91.7%). Before admission, the majority of participants reported performing activities of daily living independently (91.7%). Regarding the fracture types of the hip, most participants (49.2%) had a femoral head fracture following an intertrochanteric fracture (42.5%). Based on their risk of surgery assessment, more than half of the participants were in the American Society of Anesthesiologists (ASA) class III–IV (60%), with the remainder being in ASA class I–II (40%). The participants’ average length of hospital stays (LOS) was 12.92 ± 11.60 days (range = 4–106 days; Mean = 12.92 days, SD = 11.60 days). The participants received internal fixation (59.2%) or hip arthroplasty (40.8%) to treat their fractures. After discharge from the hospital, all participants returned to their homes, with more than one-third (86.7%) having a family caregiver to provide care. Additional details of the participant’s personal and health information are illustrated in Table 1.

### 3.2. Study Variables

Study variables included variables of interest focusing in this study. Several adverse health outcomes were identified during hospitalization (n = 42). The most common adverse health outcome was urinary tract infection (n = 24), followed by delirium (n = 23), pneumonia (n = 6), deep vein thrombosis (n = 3), and surgical site infection (n = 2). Concerning the adverse health outcomes at the two-week immediate post-hospital discharge follow-up, 16 participants had experienced adverse events, including delirium (n = 9), urinary tract infection (n = 6), surgical site infection (n = 2), and pneumonia (n = 1). However, there was no evidence of deep vein thrombosis (n = 0). Table 2 depicts the correlations between the study variables and functional ability in older people undergoing hip surgery in this study.

Regarding functional ability, the participants experienced partial recovery, as the median functional ability score was 51.41 (SD = 18.58) at the two-week immediate post-hospital discharge follow-up. Participants’ pain levels when going up or downstairs (median = 2.00, SD = 1.158) and walking on an uneven surface (median = 2.00, SD = 1.067) improved. A disability that disrupted their activities of daily living improved overall 2 weeks post-hip surgery, including activities such as rising from sitting (median = 3.00, SD = 0.993), sitting (median = 2.00, SD = 1.020), bending to the floor or picking up an object (median = 2.00, SD = 1.275), and lying-in bed (median = 2.00, SD = 1.076).

### 3.3. Predictive Power of Personal Factors and Adverse Health Outcomes on Functional Ability

The multivariate logistic regression analysis revealed that personal factors and adverse health outcomes during hospitalization and two weeks immediately post-hospital discharge could explain 28.2% (Nagelkerke R^2^ = 0.282) of the variances in functional ability in older people undergoing hip surgery.

Exploring each variable’s relationships to the outcomes in the model, the findings demonstrated that age, hip arthroplasty, and subtrochanteric fracture were the factors that were significant predictors of poor functional ability two weeks immediately post-hospital discharge (*p* < 0.05). The most noteworthy finding was that older adults with subtrochanteric fractures were 13.48 times at risk of having developed poor functional ability by two weeks after post-hospital discharge. Regarding the types of surgery, older adults who received hip arthroplasty were at 4.105 times greater risk of developing poor functional ability by two-week immediately post-hospital discharge compared to those having a surgical hip fixation. With regard to the effects of age, each one-year increase in age meant they were 1.114 times more likely to experience poor functional ability by two weeks post-hospital discharge. However, the relationships identified in this study between gender, comorbidity, adverse health outcomes during hospitalization, adverse health outcomes during two-week post-discharge, ASA class 3–4, and a fracture at the head of the femur were not significantly related to poor functional ability at two-weeks immediately post-hospital discharge (*p* > 0.05). The details of the multiple logistic regression analysis are presented in Table 3.

## 4. Discussion

According to the multiple logistic regression model, the study’s findings revealed that age, type of hip fracture, and type of surgery significantly predicted functional ability two weeks after post-hospital discharges. In terms of age, this study revealed that the majority of participants undergoing hip fracture surgery were classified as middle-old, with an average age of 78.10 years (SD = 8.37; range = 60–93 years), which is consistent with previous studies regarding the impact of aging on delayed recovery and poor functional ability after a hip surgery. A number of studies have revealed that older adults, particularly the middle-old group, experience a slight delay in functional recovery after hip surgery compared to those considered young-old [25,26,27]. Moreover, in this study, 47.5% of the participants who had hip fractures were aged 80 years or over, which is consistent with studies in the People’s Republic of China, Taiwan, and Japan, where the incidence of hip fractures increased with increasing age, especially for those aged 55 years and older [1,14,28]. The current findings add substantial evidence to the body of work indicating that aging has a considerable influence on the health and functional ability of older surgical patients, particularly those who suffer from hip fractures. Remarkably, several prior studies have noted the significance of the aging processes in stimulating age-related functional decline and physiological changes, which result in diminished musculoskeletal function. Poor musculoskeletal function leads to significant joint restriction, decreased physical activity, and increased dependency, all of which contribute to adverse health outcomes, such as fall-related injuries and immobility [29,30]. Undeniably, extensive monitoring and risk assessment are essential to the early detection of any functional deterioration in this population. Thus, effective rehabilitation and timely functional assessment are of concern to help older adults regain functional ability after hip surgery [5,6,11]. 

As for the type of hip fracture, the present study showed that subtrochanteric fractures demonstrated poor functional ability recovery two weeks after post-hospital discharge compared to other fracture types. Joseph and colleagues [31] demonstrated that patients with femoral neck fractures performed better than those with intertrochanteric or subtrochanteric fractures in the rehabilitation department. This finding might be explained. Subtrochanteric fractures were rehabilitated with weight-bearing limitations affecting functional ability [31]. Evidence also highlights that older age with a subtrochanteric fracture is a significant factor in developing substantial adverse effects on functional decline [32]. Although our findings are similar to previous studies, one of the issues arising from the findings is that the surgical goal and expected functional recovery should be prioritized, and personalized care plans should be considered for older adults with hip fractures. These findings suggested that, despite the promptness of hip surgery treatment, the types of hip fractures substantially impact the functional recovery and rehabilitation outcomes of older adults undergoing hip fracture surgery. Therefore, further research with more focus on the different anatomical types of hip fractures and functional recovery from them, in both the short and long terms, is suggested.

Regarding the type of surgery, the choice in surgical procedures for hip fractures seems to be more related to fracture type rather than age and, possibly, the general medical status of the patients. The current study found that the most common surgical procedures and rehabilitation treatments were internal fixation procedures (52.9%) and non-weight bearing after surgery (35.8%). This study’s findings revealed that internal fixation procedures of the hip were a significant predictor of poor postoperative functional ability immediately after hospital discharge. These results are consistent with previous studies [26,29]. However, internal fixation requires a longer time for the bone to heal, meaning that it may be less likely for patients to regain normal functional ability when followed up at 12 months [15]. Moreover, aging processes are a significant factor in the reduction of bone mineral density and muscle strength, which leads to poor bone health and delayed bone remodeling [1]. Although hip replacement may be beneficial in speeding up and improving postoperative functional recovery, it may increase the risk of complications and mortality [33]. The choice of surgical procedure seems controversial in older adults with hip fractures, but taking the high incidence of adverse outcomes, mortality, and long-term physical dysfunction into consideration may be essential for providing better surgical care to this population. Thus, internal hip fixation surgery might be the most practical surgical choice, as it requires a shorter time to complete the procedure and is associated with fewer perioperative complications. Notably, there is a need for additional research focusing specifically on internal fixation of hip surgery procedures and rehabilitation designed to improve functional ability recovery immediately post-hospital discharge.

In the present study, no significant associations were identified between functional ability after surgery immediately post-hospital discharge with the variables of gender, comorbidity, ASA classification, femoral head fracture, adverse health outcomes during hospitalization, and post-hospital discharge. In accordance with the previous study, gender was an insignificant predictor of functional ability [27]. Our findings regarding the prevalence of hip fractures among females are also consistent with those of numerous other studies in many countries worldwide and indicate that females are more likely to experience hip fractures than males [34,35]. The evidence highlights that the biological and physiological differences between genders might influence both bone health and functional recovery. Among females, advanced age-related post-menopausal changes may be a predisposing factor related to increased muscle weakness and poor bone health, which, in turn, leads to increased risks of developing osteoporosis, falls, and fall-related injuries [36]. Poor bone health and muscle strength have been shown to delay functional recovery and the ability to perform activities for at least one year post-hip surgery [37]. However, there was no gender-based difference in the improvement of physical ability two weeks after hospital discharge. Thus, age-related conditions may negatively influence postoperative musculoskeletal recovery during the two-week period following hospital discharge, resulting in poor physical ability in both males and females. These findings may contribute to the existing understanding of age-related conditions—a geriatric syndrome known as frailty—by highlighting the influence of potentially confounding factors that may impede the improvement of physical ability post-hip surgery in older adults. This matter requires additional study to more fully understand its implications for patient care.

Regarding comorbidities, three-four of the participants had underlying diseases, including hypertension, dyslipidemia, and type II diabetes mellitus. Comorbidity also did not predict functional ability two weeks post-hospital discharge. Our findings differ from prior studies [26,38,39], which have indicated that comorbidities affect functional recovery. Additionally, the findings of this study revealed that severe health conditions—those with an ASA classification of 3–4—did not predict good functional ability two weeks post-discharge when compared to the other classifications, which differ from the findings of previous studies [40]. 

No difference in functional ability improvement two weeks after discharge home is likely to be related to aging processes, the type of hip fracture, and the type of surgical treatment. Due to the old age nature of the participants in this study, the surgical choice seems to be based on similar concerns. Thus, the impact of the aging processes on bone remodeling and functional recovery seems to have a more profound influence than multiple comorbidities during the immediate postoperative period. One note of caution is that these findings may not be extrapolated to long-term follow-ups. Evidence showed that older people with an ASA classification of 1–2 experienced poor functional ability for one year after hip surgery [35]. Moreover, having an ASA classification of 1–2 was associated with longitudinal changes in both physical recovery and various aspects of health-related quality of life [41]. However, preventing perioperative complications may be the key to promoting functional recovery, preventing mortality, and improving health-related quality of life in both the short and long term.

Regarding the type of hip fracture, in the present study, femoral head fractures were the most common, but the data showed no significant association with poor functional ability after hip fracture surgery. Our findings support the evidence revealed in earlier investigations that there was no significant relationship between the type of hip fracture and functional ability recovery of older and more dependent [42]. Our findings support the evidence revealed in earlier investigations that there was no significant relationship between femoral head fracture and functional ability recovery [43]. These results are likely to be related to several factors—such as fracture grading, severity of injuries, and type of surgical treatment—which may impact the timeline for functional ability recovery in older adults with femoral head fractures. Fractures of the femoral head are related to the increased complexity of the injuries to the hip and its adjacent tissue areas due to the mechanisms of injury (MOI)—related to femoral head fractures generally being high—velocity energy leading to posterior dislocations and hip instability [44]. Thus, patients with a fracture of the femoral head seem to suffer from additional traumatic conditions related to the fracture site and MOI, which could mean that surgical treatment by itself may not allow them to recover functional ability in the short term. Importantly, there is no consensus on the best treatment for fully restoring functional ability post-hip surgery, particularly in older adults.

Adverse health outcomes during hospitalization and post-hospital discharge did not predict poor functional ability at immediate post-hospital discharge, which differed from prior studies [45]. In the present study, although some adverse health outcomes were documented during hospital admission, no severe adverse events that required transfer to the intensive care unit occurred. Evidence indicated that appropriate management of adverse events post-hip surgery can shorten the length of stay and enhance early postoperative rehabilitation [46], which is beneficial for fully regaining functional ability. However, another study reported that there was a significant association between adverse health outcomes—postoperative complications, infections, pneumonia, urinary tract infections, delirium, skin problems, dyspnea, and implant problems—and gait function after hip surgery [47]. Koutalos and colleagues [45] demonstrated that functional decline—the Barthel Index (BI)—was identified at a one-year follow-up, particularly worsening in the physical domain in older patients who underwent surgical treatment for hip fracture. Also, the study of Fitzgerald and colleagues [48] highlighted that the time to surgery (36 h or less after injuries) and prior injury functional status were significant factors predicting independent mobility one week post-surgery. However, these results should be interpreted with caution because the time for surgery and early ambulation interventions may influence the health outcomes in the present study because most of the participants received hip surgery within 48 h after being admitted to the hospital, and 91.7 percent were able to either move early, or perform independent activities, quickly after surgery. In addition, after discharge, the professional care teams—the home healthcare team providers—visit 3–5 days post-hospital discharge, which is a practical time for early identification of delayed recovery or complications. Thus, poor functional recovery may be less likely to occur within two weeks after post-hospital discharge. As a result, integrating preoperative care, early rehabilitation, and active functional status monitoring is essential for preventing functional decline (activities of daily living: ADLs), promoting independent mobility, and enhancing the quality of life in this population.

## 5. Strengths and Limitations

This study strengthens our understanding of recovery trajectories in older adults undergoing hip surgery that are prone to develop adverse health outcomes during hospitalization and within two weeks post-discharge. However, the data collection was collected during the COVID-19 pandemic, and the length of hospital stay for the participants varied, which may impact the functional ability of older participants. Therefore, the result of the study may not be generalized to all older people undergoing hip surgery in normal circumstances. Moreover, even though the sample size was calculated as recommended for data analysis, a larger and more diverse sample may have more advantages in increasing model prediction. In addition, the effects of walking ability on physical function after hospital discharge remain ambiguous in this study. Therefore, more research is required to investigate the clinical differences in functional outcomes among non-weight-bearing, partial-weight-bearing, and full-weight-bearing in older adults undergoing hip surgery.

## 6. Conclusions

The findings of this study demonstrated that age, type of hip fracture, and type of hip surgery significantly predicted functional ability in older adults undergoing hip surgery two weeks post-hospital discharge. The practical implications of these findings support the recommendations to strengthen the quality of care for health professionals and nurses, especially gerontological nurse practitioners and healthcare teams who work with orthogeriatric patients with hip fractures, and that they should broaden their roles regarding the early identification of these patients who are at risk of developing adverse health outcomes and initiating early rehabilitation interventions. Specific consideration of the patient’s age, anatomical type of hip fracture, and type of surgery may be beneficial for enhancing the quality of care and preventing disability after hip surgery.

## Figures and Tables

**Table 1:** Number and percentages of demographics and clinical status of the participants (N = 120).

Variables	N (%)	Variables	N (%)
**Gender**		**Smoking history**	
Female	100 (83.3)	Never smoking	108 (90.0)
Male	20 (16.7)	Used to smoke	12 (10.0)
**Age** (years) Range = 60–93 years		**Alcohol drinking history**	
Mean = 78.10; SD = 8.37		Never drinking	103 (85.8)
60–69	22 (18.3)	Used to drink	13 (10.8)
70–79	1 (34.2)	Currently drink	4 (3.3)
≤80	57 (47.5)	**ASA class**	
**Education**		1–2	48 (40.0)
No formal education	12 (10.0)	3–4	72 (60.0)
Primary school	62 (51.7)	**Type of fracture**	
Secondary school	31 (25.8)	Head	59 (49.2)
Bachelor’s degree or higher	15 (12.5)	Intertrochanteric	51 (42.5)
**Number of comorbidities**		Subtrochanteric	10 (8.3)
No	10 (8.3)	**Type of surgery**	
Yes	110 (91.7)	Arthroplasty	49 (40.8)
1–3 diseases	76 (63.3)	Fixation	71 (59.2)
>3 diseases	34 (28.3)	**Walking ability at**	
**Type of comorbidities**		**discharge**	
Hypertension	98 (81.7)	Partial weight-bearing	39 (32.5)
Dyslipidemia	67 (55.8)	Weight as tolerates	38 (31.7)
Diabetes Mellitus	43 (35.8)	Non-weight bearing	43 (35.8)
Bone and joint diseases	26 (21.7)	**Type of gait aids**	
Cardiovascular diseases	18 (21.7)	Walker	75 (62.5)
		Wheelchair	45 (37.5)
**Functional ability before admission**		**Caregivers**	
Total self-care independently	110 (90.0)	Family	104 (86.7)
Partial self-care independently	10 (10.0)	Paid	1 (0.8)
**Length of Hospital Stay** (**days)**		Nursing home	15 (12.5)
Range 4–106 days;			
Mean = 12.92 days;			
Median = 10 days; SD ± 11.6 days			

ASA class—American Association of Anesthesiologist Physical Status Classification System; SD—standard deviation.

**Table 2:** The correlation among study variables and functional ability in older people undergoing hip surgery (N = 120).

Variables	1	2	3	4	5	6	7	8	9
Gender	1.000								
2. Age ^a^	0.105	1.000							
3. Comorbidities	−0.027	−0.039	1.000						
4. ASA class	0.046	0.300 **	0.185 *	1.000					
5. Type of fracture	0.040	−0.289 **	−0.200 *	−0.012	1.000				
6. Type of surgery	0.038	−0.186 *	−0.118	−0.014	0.709 **	1.000			
7. Adverse health outcomes during hospitalization	0.141	0.271 **	0.158	0.100	−0.069	0.066	1.000		
8. Adverse health outcomes during 2 weeks post-discharge	−0.175	0.120	0.118	0.020	0.050	0.073	0.175	1.000	
9. Functional Ability	−0.142	0.207 *	0.126	−0.020	−0.022	0.071	0.128	0.190 *	1.000

Analyzed by using Spearman’s Rank; ^a^ Pearson product-moment correlation coefficient; * *p* < 0.05, ** *p* < 0.01.

**Table 3:** Multiple logistic regression analysis of personal factors and adverse health outcomes on functional ability in older people undergoing hip surgery.

Variables	B	S.E.	Wald	Df	Sig.	Exp(B)
**Gender**						
Female (reference)						
Male	1.106	0.617	3.209	1	0.073	3.023
**Age**	0.108	0.034	10.225	1	0.001	1.114
**Comorbidity**	1.566	0.871	3.227	1	0.072	4.786
**Type of surgery**						
Fixation (reference)						
Arthroplasty	1.1412	0.717	3.879	1	0.049	4.105
**Adverse health outcomes**						
During hospitalization	0.075	0.464	0.026	1	0.871	1.078
During 2 weeks post- discharge	0.735	0.685	1.153	1	0.283	2.086
**ASA class**						
Class 1–2 (reference)						
Class 3–4	−0.598	0.469	1.626	1	0.202	0.550
**Type of fracture**						
Intertrochanteric (reference)						
Head	−0.373	0.718	0.270	1	0.603	0.689
Subintertrochanteric	2.601	0.983	7.002	1	0.008	13.484

−2Log likelihood = 137.862; Chi-square = 28.460, df = 8, *p* < 0.05; Cox and Snell R^2^ = 0.211, Nagelkerke R^2^ = 0.282; Hosmer and Lemeshow Test: *p* > 0.05; Classification accuracy = 50.8%.

## Data Availability

Data are available upon request due to restrictions, e.g., privacy or ethical concerns.
